# Tracing the history of clinical practice of liquid biopsy: a bibliometric analysis

**DOI:** 10.3389/fimmu.2025.1574736

**Published:** 2025-05-13

**Authors:** Shuo Zhang, Hongwei Zhao, Kangchun Wang, Lijie Li, Qi Pan, Meitong Lu, Xing Zhang

**Affiliations:** ^1^ College of New Chinese Medicine, Nanjing University of Chinese Medicine, Nanjing, China; ^2^ Department of Third Cardre’s Ward, General Hospital of Northern Theater Command, Shenyang, China; ^3^ School of Medicine, Southeast University, Nanjing, China; ^4^ Department of Ultrasound, Liaocheng People’s Hospital, Liaocheng, China; ^5^ Department of Organ Transplantation and Hepatobiliary, The First Affiliated Hospital of China Medical University, Shenyang, China; ^6^ The Key Laboratory of Organ Transplantation in Liaoning Province, The First Affiliated Hospital of China Medical University, Shenyang, China

**Keywords:** liquid biopsy, clinical practice, biomarkers, bibliometric analysis, cancer

## Abstract

**Introduction:**

Liquid biopsy holds great promise in clinical diagnosis, treatment, and prognostic monitoring. This study reveals the development of liquid biopsy in clinical practice through a comprehensive bibliometric analysis.

**Methods:**

A total of 40 years of research literature in this field was included from the Web of Science Core Collection (WoSCC), analyzing the evolving research trends of liquid biopsy in clinical practice. We constructed co-occurrence networks for countries, institutions, authors, and keywords, integrating citation analysis and journal impact metrics to provide a comprehensive view of the research landscape in the field of liquid biopsy.

**Results:**

The results show a significant growth trend in the clinical practice of liquid biopsy, with China and the United States being the leading contributors. Institutions such as Harvard University and the University of California system play a central role in the global collaboration network. Cancers has become the primary publication outlet for the field, while highly cited journals like Clinical Cancer Research play a crucial role in advancing its development. Keyword analysis reveals that research has progressively expanded into clinical applications, personalized treatment, and prognostic evaluation.

**Discussion:**

Overall, as technology and applications continue to mature, liquid biopsy is expected to play an even greater role in the early diagnosis, treatment evaluation, and personalized treatment of cancer and other diseases.

## Introduction

Liquid biopsy, as an emerging diagnostic technology, has gained widespread attention in clinical medicine in recent years due to its non-invasive nature, high sensitivity, and ease of operation. This technology mainly involves collecting and analyzing biomarkers from body fluids such as blood, urine, cerebrospinal fluid, and saliva to help doctors achieve early disease diagnosis, personalized treatment, efficacy evaluation, and prognostic monitoring (1). The core of liquid biopsy lies in the numerous biomarkers contained in these body fluids, including circulating proteins (CP), circulating tumor DNA (ctDNA), circulating tumor RNA (ctRNA), extracellular vesicles (EVs), messenger RNA (mRNA), long non-coding RNA (lncRNA), circulating tumor cells (CTCs), and tumor-platelet emboli (TEP), among others (1–3). These biomarkers can reflect the pathological state of the body, providing important molecular information for disease detection and monitoring. For example, ctDNA can reveal the genomic characteristics of tumors, while CTCs reflect the risk of tumor metastasis. The diverse applications of liquid biopsy not only improve the accuracy of medical diagnosis but also offer patients a more convenient and safe testing method.

In early tumor screening, liquid biopsy shows great potential. Compared with traditional imaging and histological methods, liquid biopsy can detect the molecular characteristics of early tumors through ctDNA and CTCs in the blood, helping doctors identify cancer early and formulate effective interventions (4). This technology overcomes the limitations of traditional methods, especially in tumors that are difficult to diagnose via imaging, offering higher sensitivity and specificity (5). In early diagnosis, CTCs were detected in 90.5% of patients. In gastric cancer patients and healthy individuals, the sensitivity and specificity of CTC detection were 85.3% and 90.3%, respectively. Moreover, liquid biopsy demonstrated higher sensitivity in detecting advanced gastric cancer patients (6). The dynamic monitoring ability of liquid biopsy makes it an essential tool in tumor diagnosis. By obtaining molecular information from liquid biopsy, doctors can dynamically assess the tumor burden and genetic mutations of patients, providing a scientific basis for developing personalized treatment strategies. Throughout the cancer treatment process, liquid biopsy enables real-time tracking of tumor gene mutations. This dynamic monitoring ability is particularly useful in evaluating treatment efficacy and adjusting treatment plans. Cancer treatment often involves chemotherapy, radiotherapy, or immunotherapy (7–10). During treatment, by detecting changes in physical indicators or molecular markers of tumors, doctors can assess patients’ remission status and adjust treatment plans, thereby improving efficacy (11–13). This technological advantage lays a solid foundation for achieving precision medicine.

In addition, liquid biopsy plays a crucial role in monitoring disease recurrence and progression. Traditional recurrence monitoring methods often rely on regular imaging and tissue biopsy, which can be delayed and invasive (14). Liquid biopsy, on the other hand, can predict disease recurrence and progression earlier and more accurately by analyzing changes in biomarkers in body fluids. For example, after clinical treatment, CTC-positive bladder cancer patients have poorer progression-free survival, cancer-specific survival, and overall survival (15, 16). CTCs can be used to assess the efficacy of cisplatin chemotherapy, PD-L1 immunotherapy, and other treatments, helping to better predict treatment outcomes. Compared with CTC-negative patients, CTC-positive patients have higher cancer-related mortality and disease recurrence rates. CTC-positive patients receiving neoadjuvant chemotherapy have a longer survival time than non-CTC-positive patients (17).

With continuous technological advances, liquid biopsy has undergone multiple breakthroughs. Early development issues, such as low detection sensitivity and high false positive/false negative rates, are gradually being resolved. Liquid biopsy represents a paradigm shift in diagnostics. Blood has traditionally been a primary target for biopsy due to its accessibility and rich content of diverse biomarkers. However, the applications of liquid biopsy are rapidly expanding beyond blood-based assays. For instance, cerebrospinal fluid can be utilized for liquid biopsy in brain cancer, breast milk may serve as a medium for breast cancer detection, and pleural effusions can be analyzed for thoracic tumors or chronic diseases (3). The field has also transitioned from basic research to clinical application, and understanding its development trajectory will help promote more effective translational medicine research. Bibliometric analysis, by quantitatively analyzing keywords, research topics, and author collaboration networks in scientific literature, can reveal the development trends, research hotspots, and knowledge maps of a particular field (18). This study used bibliometric analysis to conduct a comprehensive analysis from multiple dimensions, including global publication trends, cooperation networks between researchers and countries, the most influential journals and papers in the field, and keyword analysis, to explore the development trends and technological evolution in this area. In conclusion, this study conducted a comprehensive bibliometric analysis of the historical development, current research status, and future potential of liquid biopsy in clinical applications.

## Methods

### Data collection

The Web of Science Core Collection (WoSCC) covers a broad range of literature across multiple academic disciplines, supports analysis using various bibliometric software tools, and is the most widely used database in the field of bibliometrics. Therefore, WoSCC serves as the primary data source for analysis in this study. To ensure data accuracy and completeness, the WoSCC database search was completed on October 22, 2024. All search operations were independently performed by two staff members on the same day to ensure consistency and reliability of the data. The search results were downloaded in two formats: “Full Record and Cited References” and “Plain Text,” for ease of subsequent analysis and processing. The search strategy used was: TS = (liquid biops*) AND (‘diagno*’ OR ‘therap*’ OR ‘prognos*’ OR ‘detection’ OR ‘screening’ OR ‘treatment’ OR ‘disease management’ OR ‘survival’ OR ‘clinical’).

Using this strategy, we successfully downloaded 14,859 records from 1985 to 2024 that met the search criteria. During the data cleaning and screening phase, irrelevant entries such as conference papers, review articles, reprints, retractions, and corrections were removed, and the language was restricted to English, resulting in 9,232 valid references. Additionally, all downloaded data were carefully checked to ensure there were no duplicate entries.

### Data analysis

The data management and bar chart generation for this study were conducted using Microsoft Excel 2024, enabling effective organization and visualization of the data. The Bibliometrix R package (version 4.3.0), specifically designed for bibliometric analysis, was used to process, analyze, and visualize information from the literature database. During the analysis, an interactive web application based on the Shiny framework was used to examine trends in international collaboration, author and journal publication counts, and citation patterns over time. VOSviewer (version 1.6.20) was used for bibliometric analysis and scientific network visualization. In this study, it was employed to construct relationship networks between international collaborations, institutions, authors, and keywords. All data were derived from WoSCC’s plain text files, with full counting applied for the analysis. Based on VOSviewer’s analysis results, the Scimago Graphica tool (version 1.0.45) was used to create a country collaboration network, illustrating the cooperation between different nations. Microsoft Charticulator was also used to visualize the author collaboration network. CiteSpace (version 6.4.R1 Advanced) is a Java application used to visualize collaboration networks and the evolution of research trends within a particular field. Using CiteSpace, we conducted detailed analyses of the keywords and source journals in the field, including co-occurrence analysis, timeline analysis, burst detection, and journal dual-map overlay analysis.

## Results

### Global publishing trends and international collaboration

**Figure 1:**
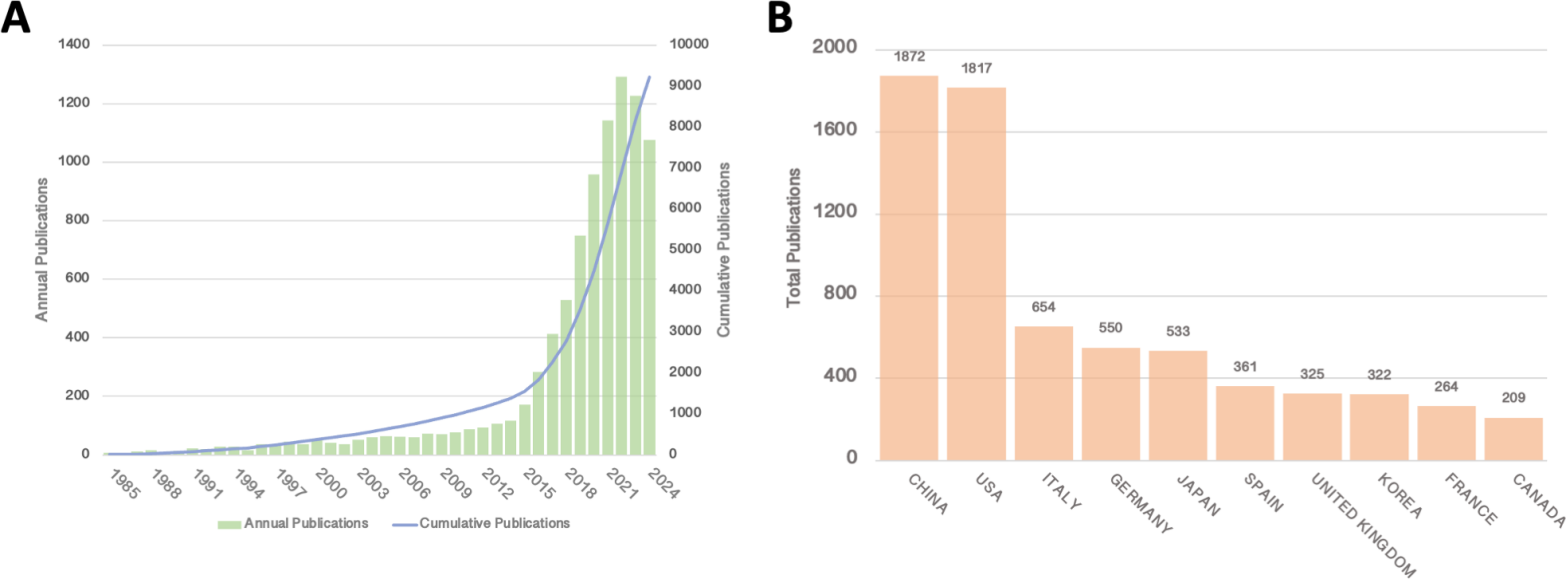
**(A)** Annual and cumulative publications from 1985 to 2014; **(B)** Top 10 countries/regions by total publications.

The number of publications related to clinical practice in liquid biopsy has steadily increased over time, with the total number of publications in the past four years surpassing that of the previous 36 years (**Figure 1A**). The results reveal two distinct phases in the development of liquid biopsy research. The early phase, spanning from 1985 to 2014, lasted for 30 years and saw a relatively slow yet steady growth in the number of publications, with annual publications peaking at around 100. In contrast, the rapid development phase began in 2015 and continues to the present, with publication numbers stabilizing in the past three years. Since 2015, the surge in publication numbers has indicated a significant increase in both interest and progress in liquid biopsy research, driven by technological breakthroughs and the growing recognition of its potential in clinical settings. The stabilization of publication numbers in recent years may reflect the maturation stage at which basic research is integrating into clinical practice.

In terms of total publication output by country, China and the United States have emerged as clear leaders in liquid biopsy research, with China publishing 1,872 articles and the U.S. following closely with 1,817 (**Figure 1B**). Several European countries and Japan follow behind. The leading position of China and the U.S. in this field can be attributed to several factors, including substantial research funding, strong technological infrastructure, and government support for medical research, which have all contributed to their prominence in liquid biopsy research. The large number of publications from these countries highlights their central role in advancing this research globally.

**Figure 2:**
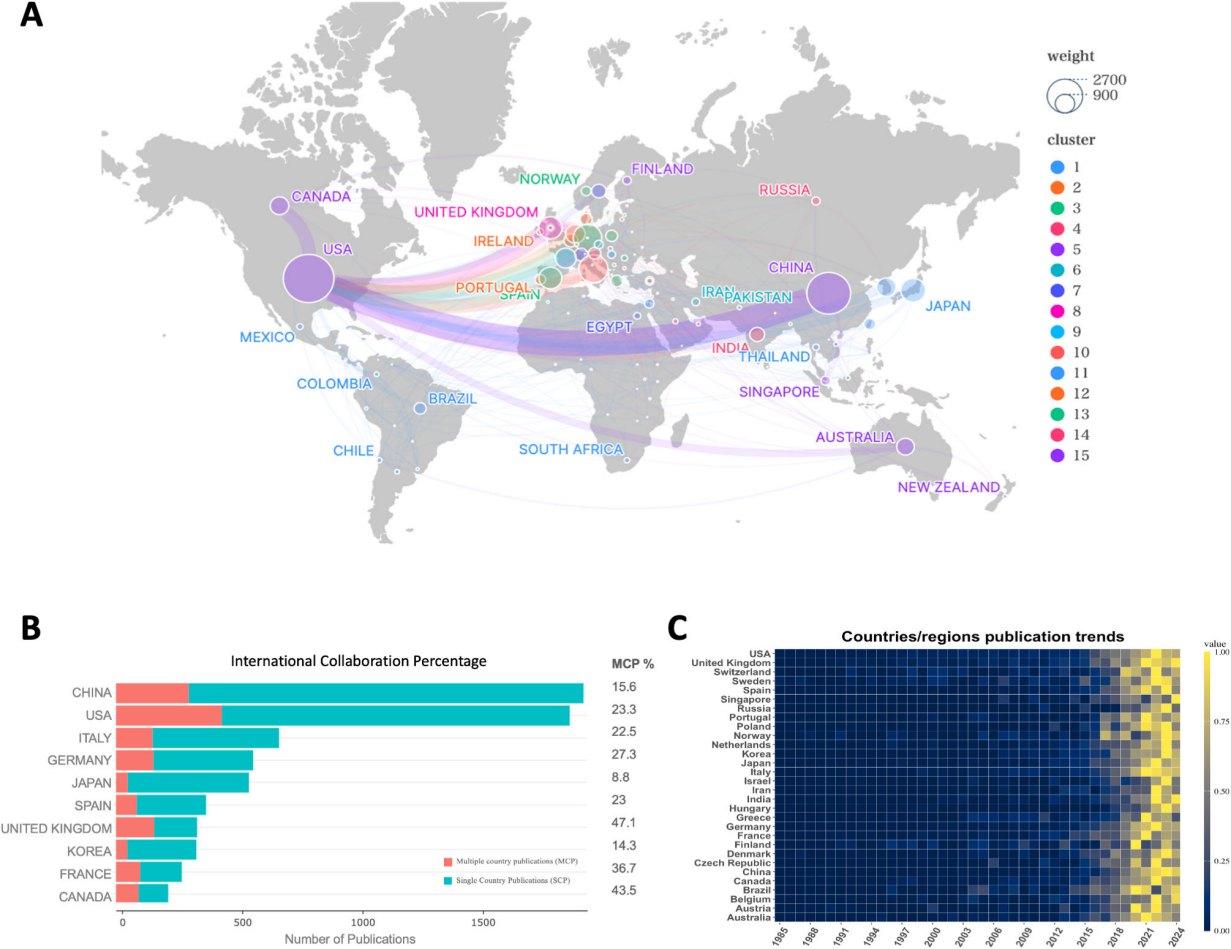
**(A)** Network of country/region collaborations; **(B)** International collaboration percentage of the top 10 countries/regions by publications; **(C)** Time heatmap of publication trends for each country/region.

Interestingly, while both China and the U.S. possess robust international collaboration networks, China’s network is more concentrated, primarily focusing on collaboration with the U.S., whereas the U.S. has a broader range of international partnerships. In addition to collaborating with China, the U.S. also works with Canada, European countries, and Australia (**Figure 2A**). In addition, publications from the United States also lead in citation counts (**Table 1**). This broader international collaboration reflects the U.S.’s greater integration with the global scientific community. The percentage of international collaborations further emphasizes this trend, with the chart illustrating the proportion of international collaborations in publications by country (**Figure 2B**). Despite China and Japan ranking high in publication volume, only 15.6% and 8.8% of their publications, respectively, are the result of international collaborations. In contrast, countries such as the U.S., Italy, Germany, Spain, the U.K., France, and Canada, while having lower total publication numbers, show a higher proportion of internationally collaborative publications, which may be partly influenced by geographical factors. Finally, the heatmap further highlights the recent surge in publications, particularly since 2015, aligning with the identified rapid development phase (**Figure 2C**).

**Table 1:** Top 15 most cited countries.

Country	Total citations	Average article citations
USA	63809	35.1
CHINA	32215	17.2
ITALY	15804	24.2
UNITED KINGDOM	14692	45.2
GERMANY	11851	21.5
JAPAN	10096	18.9
FRANCE	8041	30.5
CANADA	7431	35.6
SPAIN	6590	18.3
NETHERLANDS	5435	27.7
KOREA	4803	14.9
AUSTRALIA	4166	20.3
AUSTRIA	4058	45.1
SWEDEN	3606	34
BELGIUM	3157	29.5

### Key institutions and leading authors

Studying the collaboration networks of authors and institutions in the field of liquid biopsy clinical practice is crucial for understanding its development trends and identifying the most influential contributors. By analyzing these networks, we can gain a better understanding of the dynamics of research collaboration, the relationships between different participants, and how these interactions shape the development of the field.

**Figure 3:**
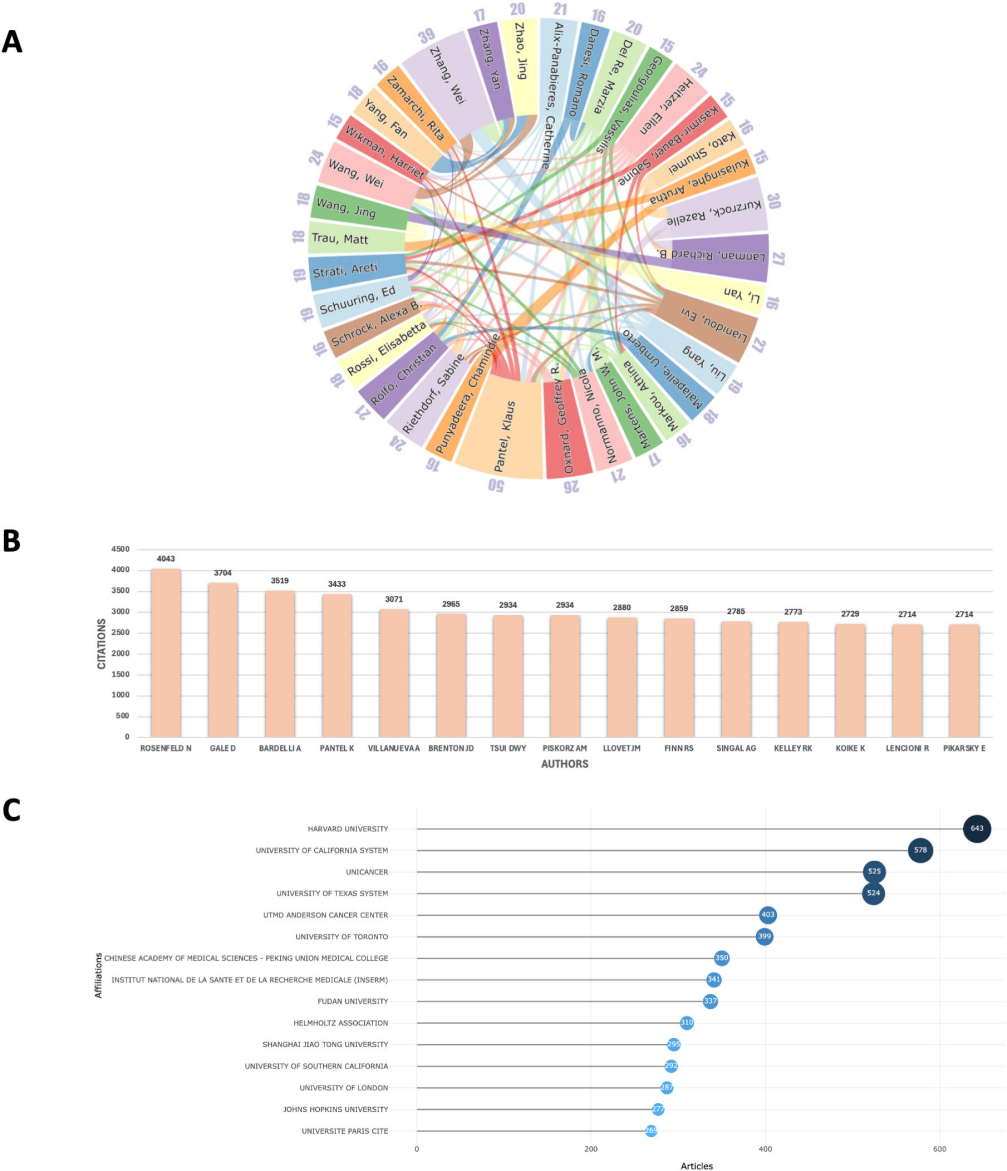
**(A)** Collaboration network of the top 35 authors in publications; **(B)** The top 15 most-cited authors; **(C)** Ranking of the top 15 institutions in publications.

In the author collaboration network, we examined the relationships among the top 35 authors with the highest publication outputs (**Figure 3A**), who represent the leading scholars in the field of liquid biopsy. Professor Klaus Pantel from Germany, with 50 publications in this area, is widely regarded as a pioneer of liquid biopsy. He has also made significant advancements in areas such as cancer micro-metastasis, CTCs, and circulating nucleic acids, including ctDNA and microRNAs. The collaboration network also shows that high-output authors tend to have closer professional connections, with Pantel collaborating with nearly a quarter of the other top authors, further consolidating his central role in the field. Furthermore, focusing solely on high-output authors may overlook influential researchers who have made significant contributions to the field. To address this, we also identified the 15 most-cited scholars in liquid biopsy (**Figure 3B**). Among them are Nitzan Rosenfeld (UK, n = 4043), Davina Gale (UK, n = 3704), and Alberto Bardelli (Italy, n = 3519), who have played crucial roles in advancing the clinical applications of liquid biopsy. Their work continues to be widely cited, highlighting their lasting impact on the field.

**Figure 4:**
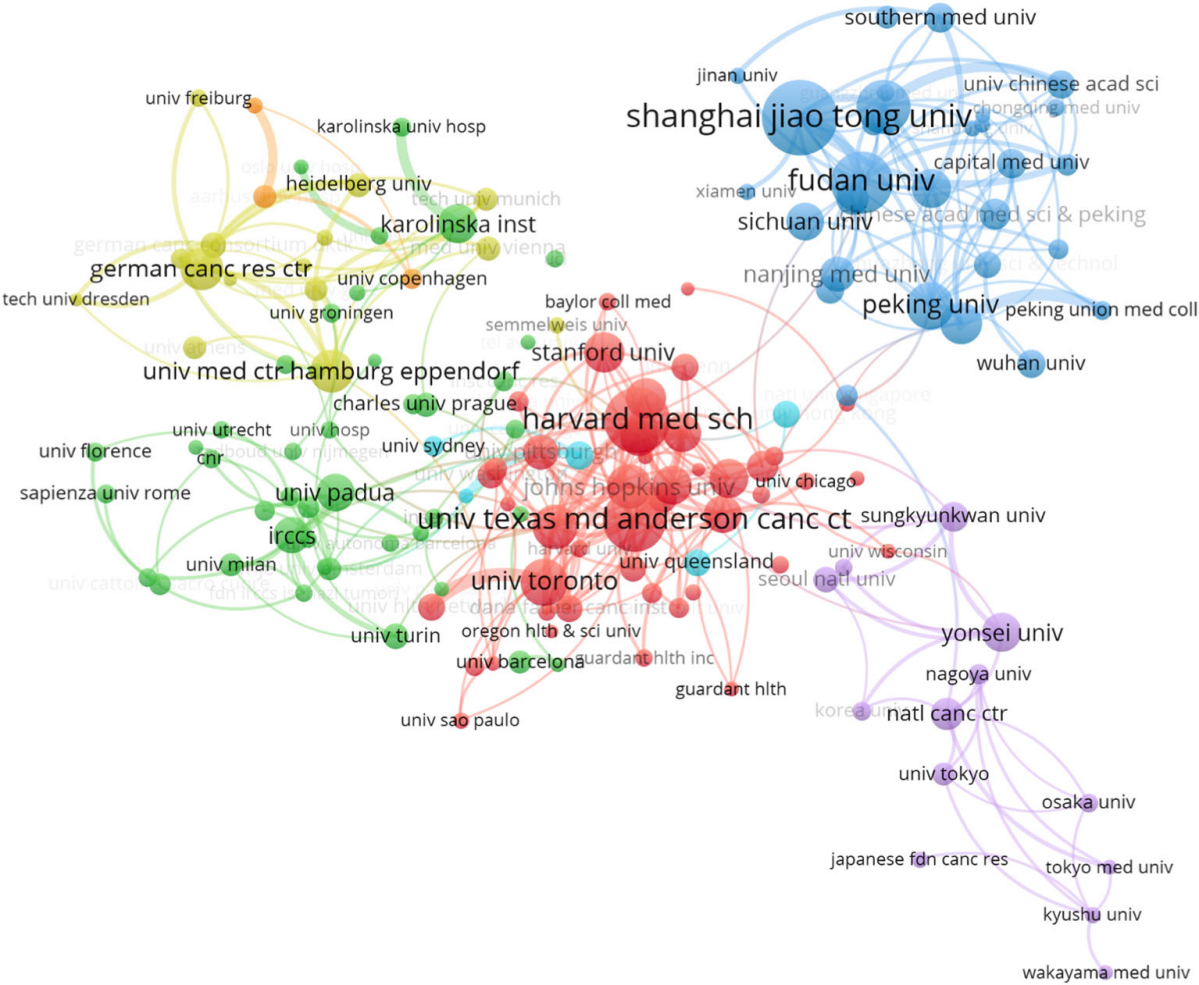
Institutional collaboration network.

In terms of institutional collaboration, we analyzed the relationships among institutions with at least 30 publications, selecting 152 institutions globally (**Figure 3C**). Among these, Harvard University emerged as the leading institution with the highest publication output (n = 643), followed by the University of California system (n = 578). Other influential institutions include Unicancer in France (n = 525), MD Anderson Cancer Center in the U.S. (n = 403), the University of Toronto in Canada (n = 399), and Peking Union Medical College in China (n = 350). These institutions represent major research hubs, each making significant contributions to the development of liquid biopsy technologies and their clinical applications. The collaboration network among these institutions reflects a clear geographic pattern, with distinct sub-networks emerging in regions such as China, the U.S., Japan, and Europe. Within these regions, collaboration is generally more frequent and closer, while larger institutions like Harvard tend to establish international partnerships, further promoting the global nature of liquid biopsy research (**Figure 4**).

Overall, the analysis of the author and institutional collaboration networks in the field of liquid biopsy clinical practice reveals a dynamic and interconnected research landscape. The close cooperation among top authors and institutions is vital for pushing the boundaries of liquid biopsy technologies and integrating them into clinical practice.

### Journal impact and highly cited publications

The analysis of journal impact provides critical insights into the development and current state of liquid biopsy research. As a cutting-edge technology, liquid biopsy has achieved rapid advancements. By identifying where the most influential works in this field are published, we can gain a clearer understanding of its developmental trajectory and the journals that have driven this progress.

**Figure 5:**
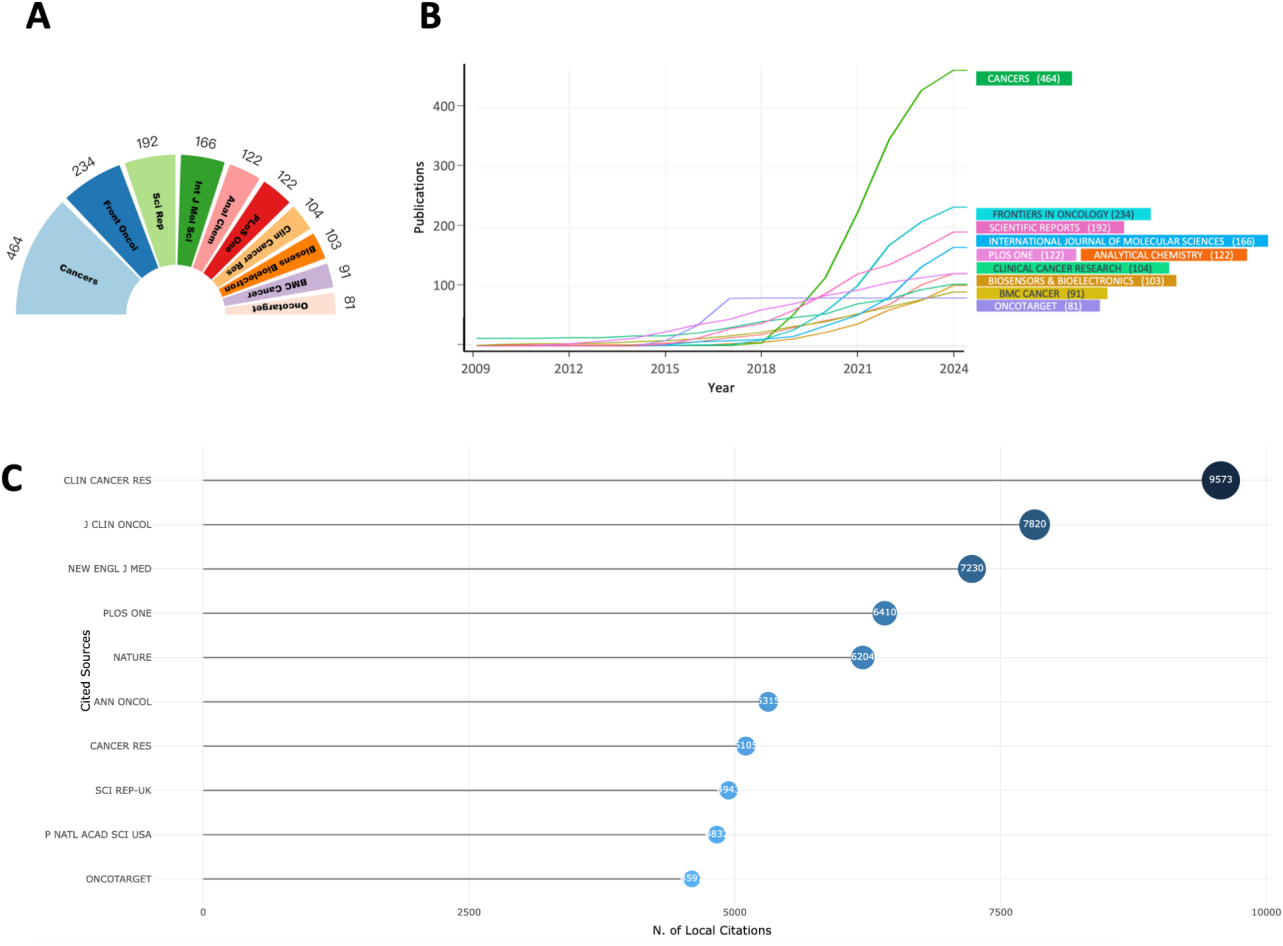
**(A)** Top 10 journals in publications; **(B)** Temporal trend of the top 10 journals in publications; **(C)** Top 10 most-cited journals;.

A detailed analysis of the top ten peer-reviewed journals with the highest publication volumes over the past 40 years (**Figure 5A**) reveals that *Cancers* (n = 464), *Frontiers in Oncology* (n = 234), and *Scientific Reports* (n = 192) are the primary contributors. Over the past 15 years, a line chart (**Figure 5B**) illustrates how publication trends have evolved. Notably, *Cancers* has experienced significant growth over the past five years, firmly establishing its leadership in this domain. This rapid increase likely reflects the journal’s growing ability to publish high-quality, impactful research. Importantly, the rise in publication volume coincides with the increasing clinical adoption of liquid biopsy technologies, such as ctDNA and CTCs, underscoring the journal’s alignment with the priorities of the field. While publication volume indicates productivity, true influence lies in the quality and impact of the research. A focus on citation metrics demonstrates that highly cited journals are often the main drivers of innovation and dissemination in the field. For instance, despite their relatively lower publication volumes, *Clinical Cancer Research* (n = 9573), *Journal of Clinical Oncology* (n = 7820), and *The New England Journal of Medicine* (n = 7230) are the most cited journals (**Figure 5C**, **Table 2**). These journals consistently publish high-caliber studies that define key milestones in the clinical application of liquid biopsy.

**Table 2:** Top 15 most cited journals.

Journal	H-index	G-index	M-index	Total citations	Number of publications
*CLINICAL CANCER RESEARCH*	45	74	1.607	5668	104
*ONCOTARGET*	42	61	4.2	4064	81
*SCIENTIFIC REPORTS*	36	56	3.6	4147	192
*ANNALS OF ONCOLOGY*	34	47	2.833	4497	47
*BIOSENSORS & BIOELECTRONICS*	33	51	2.75	2928	103
*PLOS ONE*	32	57	2.286	3729	122
*CANCERS*	31	39	2.818	5147	464
*CLINICAL CHEMISTRY*	31	48	0.775	2489	75
*CANCER CYTOPATHOLOGY*	30	45	1.071	2284	72
*PROCEEDINGS OF THE NATIONAL ACADEMY OF SCIENCES OF THE UNITED STATES OF AMERICA*	28	31	0.7	3715	31
*ANALYTICAL CHEMISTRY*	27	44	1.5	2464	122
*INTERNATIONAL JOURNAL OF CANCER*	25	40	1.19	1758	67
*LAB ON A CHIP*	25	49	1.786	2421	53
*NATURE COMMUNICATIONS*	24	49	2.182	2822	49
*BMC CANCER*	22	41	1.294	1905	91

Additionally, to determine the specific contributions of individual studies, we analyzed the top 15 most-cited publications in the liquid biopsy field (**Table 3**). Many of these seminal works were published about five years ago, providing ample time to accumulate significant citations. However, recent publications have also achieved exceptional citation performance. For example, the 2021 study by Josep et al. (19) has been cited over 2,700 times. Such studies often address broad, clinically relevant questions and provide innovative guidance, making them indispensable references for subsequent research and clinical implementation.

**Table 3:** Top 15 most cited publications.

Paper	DOI	Total citations	Total citations per year	Normalized total citations
LLOVET JM (19), NAT REV DIS PRIMERS	10.1038/s41572-020-00240-3	2714	678.5	148.81
CHENG AL (59), ANTICANCER RES	N/A	1747	72.79	19.91
DIAZ LA (60), J CLIN ONCOL	10.1200/JCO.2012.45.2011	1633	148.45	23.2
GOOTENBERG JS (61), SCIENCE	10.1126/science.aaq0179	1531	218.71	30.82
MURTAZA M (62), NATURE	10.1038/nature12065	1312	109.33	25.65
FORSHEW T (63), SCI TRANSL MED	10.1126/scitranslmed.3003726	1006	77.38	21.17
JOURA EA (64), NEW ENGL J MED	10.1056/NEJMoa1405044	971	97.1	15.97
NEAL JT (65), CELL	10.1016/j.cell.2018.11.021	868	124	17.48
GANDARA DR (66), NAT MED	10.1038/s41591-018-0134-3	848	121.14	17.07
MAYRAND M (67), NEW ENGL J MED	10.1056/NEJMoa071430	794	44.11	14.4
HOSHINO A (68), CELL	10.1016/j.cell.2020.07.009	717	143.4	32.08
IGNATIADIS M (69), NAT REV CLIN ONCOL	10.1038/s41571-020-00457-x	658	164.5	36.08
NAUCLER P (70), NEW ENGL J MED	10.1056/NEJMoa073204	623	34.61	11.3
MOULIERE F (71), SCI TRANSL MED	10.1126/scitranslmed.aat4921	616	88	12.4
GROSS S, (72), J EXP MED	10.1084/jem.20092506	608	40.53	12.68

**Figure 6:**
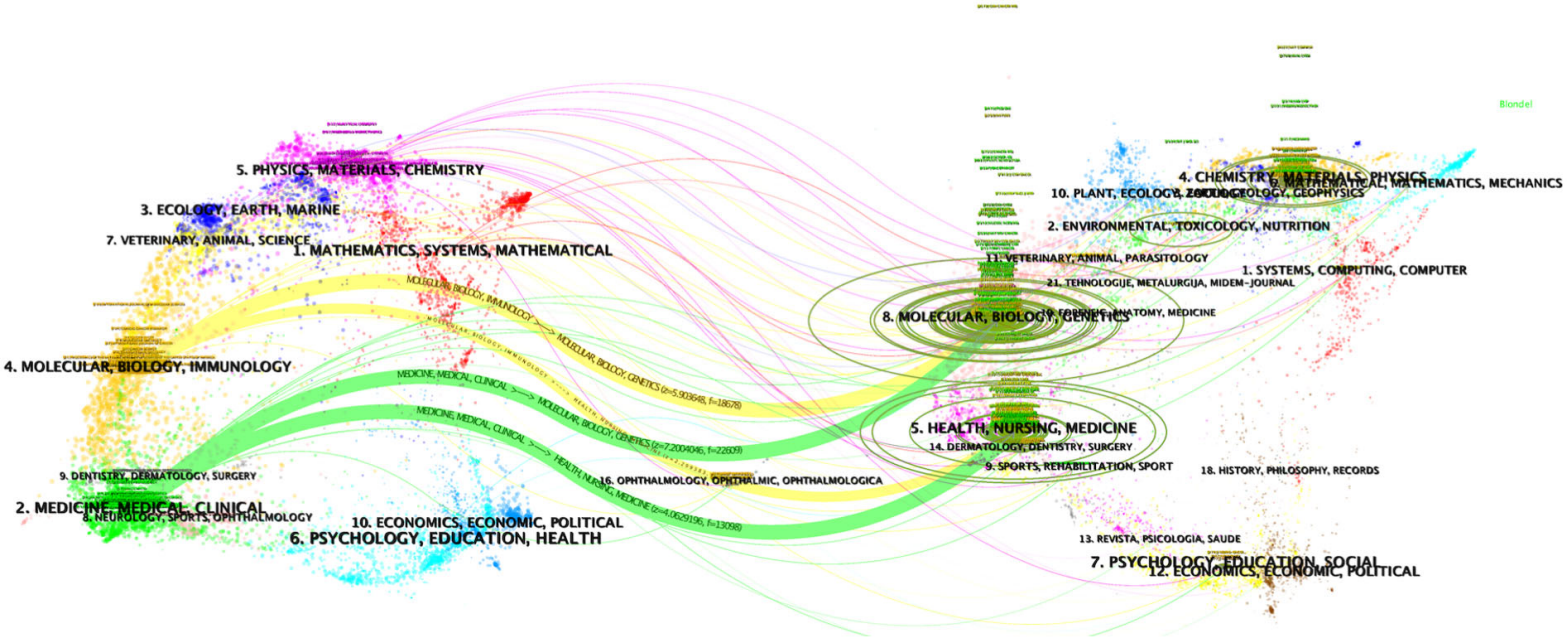
Dual-map overlay of the journals.

Finally, a dual-map overlay analysis offers a comprehensive visualization of the citation relationships and thematic connections between journals (**Figure 6**). This tool highlights the pathways through which knowledge flows in the field, revealing the multidisciplinary nature of liquid biopsy research. As the field continues to evolve, fostering interdisciplinary collaboration and prioritizing the dissemination of clinically relevant findings are essential to ensure that research translates into tangible benefits for patients. Reflecting on the development of liquid biopsy in clinical practice, it is evident that the field has transitioned from experimental techniques to a cornerstone of precision oncology. This progress has been fueled by the convergence of technological innovations, such as next-generation sequencing and bioinformatics, with clinical demands for early detection, minimal residual disease monitoring, and resistance prediction. Journals that prioritize interdisciplinary approaches and high-quality studies have played a critical role in this transformation.

### Keyword analysis and research development trends

To thoroughly analyze the research trends and development trajectory in the clinical practice of liquid biopsy, we conducted a keyword co-occurrence network analysis and a keyword burst analysis. These methods were used to identify key research themes, assess their temporal evolution, and highlight emerging areas of interest.

**Figure 7:**
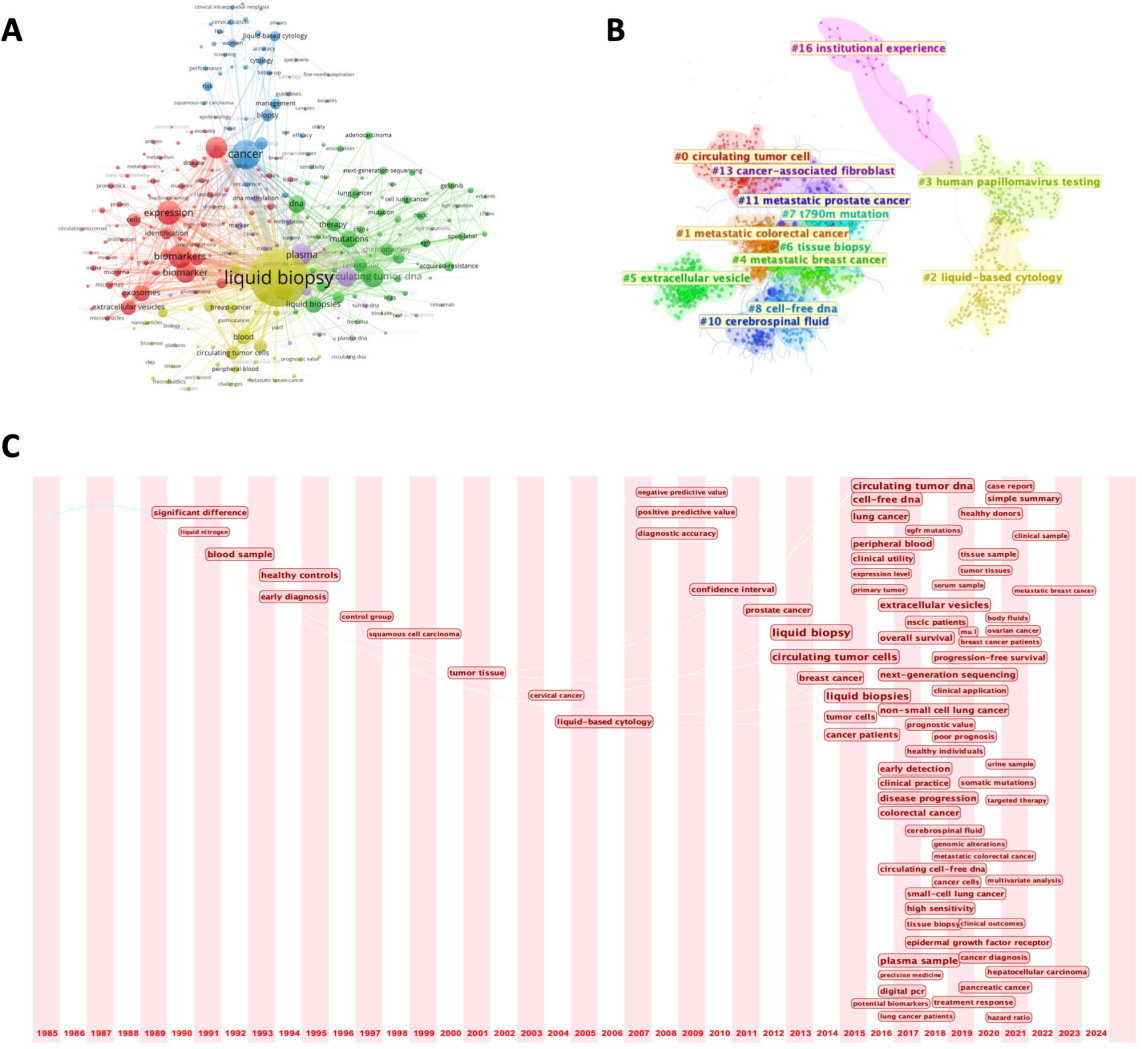
**(A)** Keyword co-occurrence network; **(B)** Keyword clustering map; **(C)** Timeline view of keywords from 1985 to 2024.

In the keyword network, we set a minimum occurrence threshold of 50, resulting in the inclusion of 252 keywords (**Figure 7A**). The term “liquid biopsy” emerged as the highest-weighted keyword, confirming its central role in the literature. Other frequently appearing terms, such as “circulating tumor DNA,” “circulating tumor cells,” and “exosomes,” highlight the primary molecular markers studied in liquid biopsy research. The prevalence of the term “cancer” indicates that oncology is the dominant application area, addressing cancers such as lung, cervical, and breast cancers. Additionally, terms like “diagnosis,” “therapy,” and “risk” reflect the diverse clinical contexts in which liquid biopsy is applied, including diagnostics, therapeutic monitoring, and prognostic evaluation.

Further analysis categorized the keywords into more than 10 distinct research clusters, each with a specific focus area (**Figure 7B**). Notable clusters include metastatic tumors, a significant area of research due to their association with abundant biomarkers (e.g., CTCs) in the bloodstream, and HPV detection, where liquid biopsy has demonstrated significant utility—particularly in reproductive system tumors. For instance, a study involving 60 cervical cancer patients confirmed that circulating cell-free HPV DNA holds promise as a prognostic biomarker (20). These clusters span a wide range of topics, from the development of novel detection technologies to non-cancer applications, such as early detection of viral infections and autoimmune diseases. This categorization highlights the breadth of research directions in the field and its expanding applications beyond oncology.

**Figure 8:**
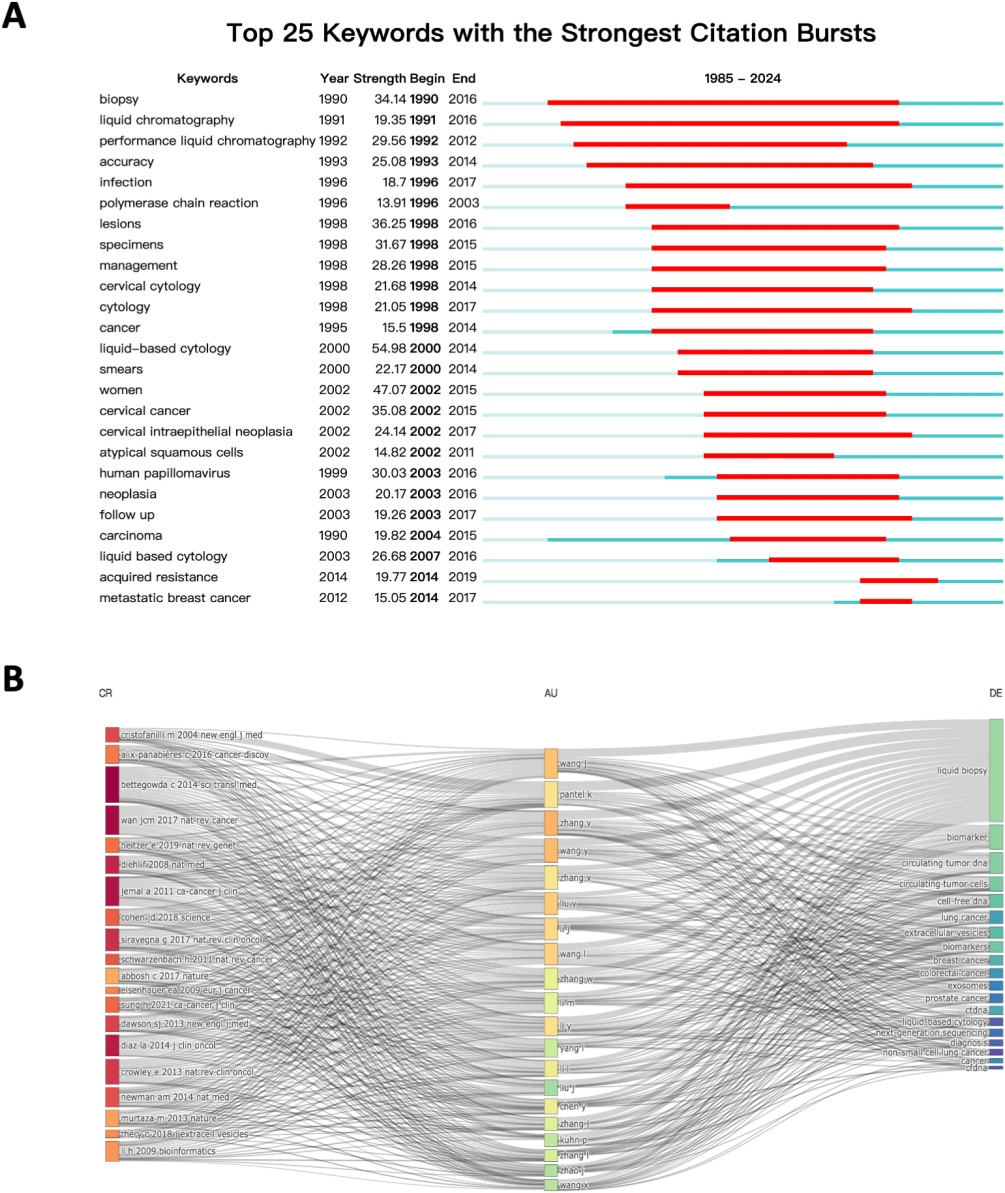
**(A)** Keyword burst analysis results from 1985 to 2024; **(B)** Sankey diagram of papers, authors, and keywords.

To examine the evolution of research priorities, we plotted a 40-year timeline of keyword occurrences (**Figure 7C**). Early studies focused on the basic analysis of blood samples and early diagnostic techniques. The term “liquid biopsy” gained prominence in 2012, marking a turning point in the field. During this period, the throughput of next-generation sequencing (NGS) technologies (such as Illumina’s HiSeq platform) significantly increased, while costs substantially decreased, making large-scale detection of ctDNA or exosomes in blood possible. Since then, advancements have included the integration of NGS and bioinformatics tools, facilitating more precise detection of minimal residual disease (MRD) and tracking of tumor evolution. Recent studies have diversified into exploring new tumor types and advanced applications such as survival prediction and targeted therapy. Keyword burst analysis aligned with this trend, with terms like “follow up” and “acquired resistance” indicating a growing emphasis on integrating liquid biopsy into routine clinical practice (**Figure 8A**). Updates in clinical guidelines have also influenced developments in this field. For instance, the first IASLC position paper on liquid biopsy in 2018 has, to some extent, altered treatment decision-making for advanced non-small cell lung cancer (21). In recent years, the prominence of keywords such as “treatment response” and “targeted therapy” reflects significant transformations in the field of cancer treatment. With the rapid advancement of targeted therapy and immunotherapy, liquid biopsy has emerged as an increasingly critical tool in precision oncology. In targeted therapy, liquid biopsy can rapidly identify therapeutic targets and detect acquired resistance mutations, guiding treatment adjustments. In immunotherapy, the analysis of ctDNA and immune-related biomarkers can predict treatment response, evaluate efficacy, and monitor immune-related adverse effects. Finally, a three-field plot was constructed to map relationships between influential papers, prominent authors, and high-frequency keywords (**Figure 8B**). This visualization provided insights into impactful research, collaborations, and emerging research foci, serving as a guide for future investigations.

In summary, the bibliometric analysis highlights the dynamic growth of liquid biopsy from a niche research topic to a multifaceted clinical tool. As liquid biopsy matures, its integration into routine care across diverse medical disciplines could significantly improve early detection, treatment monitoring, and patient outcomes worldwide.

## Discussion

Liquid biopsy is a novel disease detection method that has developed in recent years. It achieves disease diagnosis, monitoring, and treatment evaluation by analyzing biomarkers in body fluids, such as ctDNA, CTCs, and exosomes. This article provides a comprehensive bibliometric analysis exploring the global development trends and key research features of liquid biopsy in clinical practice. We focus on the research contributions of major countries, with China and the United States being core drivers of liquid biopsy research due to their high paper output. Additionally, the analysis of international collaboration networks highlights the differences in scientific cooperation among countries. The article analyzes institutional and individual contributions, identifying high-productivity research centers and prominent scholars while demonstrating how concentrated scientific resources and collaborative networks serve as key drivers of technological innovation. Furthermore, through co-occurrence and burst analysis of keywords, the article identifies key research hotspots and their evolutionary trends in the liquid biopsy field, covering diverse clinical applications ranging from early diagnosis and treatment monitoring to resistance evaluation. In conclusion, this article uses a multidimensional analysis method to comprehensively review the current research status, key contributors, and development directions of liquid biopsy in clinical practice, while emphasizing the importance of interdisciplinary integration and international collaboration in advancing technology and clinical applications.

The research on liquid biopsy dates back to before the 1990s when scientists discovered that DNA fragments released by tumor cells enter the bloodstream, laying the theoretical foundation for liquid biopsy research. In 1948, Mandel and Metais first discovered free nucleic acid molecules in plasma, providing the basis for the concept of liquid biopsy (22). However, these early studies were largely descriptive in nature and did not form systematic theories or applications. With advancements in molecular biology, liquid biopsy research gradually deepened. For example, the introduction of polymerase chain reaction (PCR) technology enabled scientists to detect cancer-related gene mutations in blood, while the isolation and functional studies of CTCs and exosomes revealed substantial information contained in liquid samples. Subsequently, liquid biopsy entered the industrial growth phase, driven by the combination of clinical demand and technological commercialization (23–25). For example, in 2005, CTCs were proven to be an important indicator for predicting the survival of breast cancer patients (26); in 2008, BEAMing technology further verified that dynamic changes in ctDNA could reflect tumor burden (27). The success of such research brought liquid biopsy from the laboratory to the clinic, showcasing its potential in cancer screening, diagnosis, and treatment monitoring. Currently, liquid biopsy has entered a phase of industry boom, fueled by regulatory support and widespread industry recognition. In 2014, the European Medicines Agency approved ctDNA for EGFR mutation detection, marking the official entry of liquid biopsy into clinical practice. In recent years, various liquid biopsy indicators, including CTCs and ctDNA, have been incorporated into cancer treatment guidelines, such as the recognition of CTCs by AJCC and CSCO, making them an important component in prognosis evaluation for diseases like breast cancer (28, 29).

The advancement of cutting-edge technologies has also propelled the clinical application of liquid biopsy. Pahattuge et al. (30) introduced a modular microfluidic system called SMART-Chip, which significantly reduces processing time by over 50% compared to manual sample handling when analyzing blood samples collected from cancer patients. Traditional ctDNA detection methods primarily rely on PCR. However, recent advances in PCR and sequencing technologies have enabled alternative approaches, including quantitative PCR (qPCR), digital PCR (dPCR), droplet digital PCR (ddPCR), and NGS. qPCR offers faster turnaround times, improved reproducibility, and better quantification by monitoring DNA amplification in real time. NGS provides broad advantages, such as screening for unknown mutations, structural variations, and copy number alterations. dPCR/ddPCR partition DNA samples into thousands or millions of compartments/droplets, significantly reducing background noise and enabling detection of tumor DNA with variant allele frequencies as low as 0.1%. Additionally, novel integrated detection strategies incorporating gene-editing technologies, functional enzymes, and nanomaterials have been developed in recent years. These approaches enhance the effective concentration of mutant fragments, thereby improving the identification of target gene mutations in ctDNA (31–34).

The development history of liquid biopsy can be divided into four main stages, each marking significant progress and transformation in the field. The initial stage, spanning the pre-1990s period, represents a phase of scientific exploration. Research during this era primarily focused on fundamental science, investigating the feasibility of detecting disease-related biomarkers in liquid samples. The second stage is the scientific development period, occurring in the 1990s, when researchers conducted preliminary trials and technological improvements for practical applications. The third stage is the industry growth period, from 2000 to 2010, during which liquid biopsy began to gradually enter the market with the maturation of technology and optimization of related equipment. The fourth stage is the industry boom period, from 2010 to the present, during which liquid biopsy technology has developed rapidly, and its clinical applications have expanded, driving the field into a stage of large-scale commercialization (35). From the development history, it is evident that liquid biopsy has progressed from basic research to clinical application, which is not only the result of technological breakthroughs but also the product of coordinated efforts between scientific research, industry promotion, and clinical demand.

In the clinical application of early diagnosis, compared to traditional cancer screening methods, such as imaging and tissue biopsy, liquid biopsy can detect tumor-related biomarkers in blood, urine, or other body fluids when the tumor is still in its early or microscopic stage, thus improving the sensitivity and specificity of early diagnosis (36). This advantage provides crucial support for early cancer detection and treatment, with the potential to significantly improve patient prognosis and survival rates. The core principle of liquid biopsy lies in detecting biomarkers such as ctDNA, CTCs) and EVs in body fluids. For example, ctDNA can reflect the genetic information released into body fluids during tumor cell proliferation and apoptosis. Its mutation frequency and types can provide insights into tumor genetic variations and malignancy, offering a reliable basis for early cancer diagnosis and typing (37). Studies have shown that liquid biopsy has achieved significant results in early screening for various malignancies, including lung cancer, breast cancer, and colorectal cancer. In these cancer types, even when the tumor is still clinically asymptomatic or of small size, liquid biopsy can sensitively detect DNA mutations or gene rearrangement signals released by cancer cells, enabling early detection and supporting subsequent treatment decisions. In addition to detecting ctDNA, liquid biopsy can further enhance early diagnostic efficacy by analyzing CTCs, EVs, and other novel biomarkers. For example, research has found that the exosome proteins LG3BP and PIGR play a key role in tumor transformation, invasion, and proliferation, while being closely associated with poor prognosis in patients. Compared to traditional markers such as alpha-fetoprotein (AFP), LG3BP and PIGR show higher sensitivity and specificity in early diagnosis of liver cancer (38, 39). Furthermore, the application of miRNA in liquid biopsy has also gained significant attention. A study showed that the combination of miRNA and AFP significantly improved diagnostic performance, especially for patients with low AFP expression (AUC: 0.80, specificity: 95%, accuracy: 81%) (40). In the early diagnosis of pancreatic cancer, liquid biopsy has shown great potential. Although traditional marker CA19–9 is widely used in pancreatic cancer detection, its diagnostic efficacy remains inadequate in early stages. By analyzing various miRNAs, studies have found that 66.10% of miRNAs outperform CA19–9 in diagnostic value (41). Based on these findings, the 2023 Expert Consensus on Early Molecular Diagnosis of Pancreatic Cancer recommended miRNA combinations as important markers for early precise diagnosis of pancreatic cancer, and their combined use with CA19–9 can further enhance diagnostic efficacy (35). Liquid biopsy, as a non-invasive testing method, offers significant advantages over traditional tissue biopsy, reducing patient trauma and discomfort caused by surgical sampling. It is particularly suitable for diagnosing patients with weak physical conditions or difficult-to-access lesions, and this flexibility provides unique value in early cancer screening and follow-up management.

In addition to early diagnosis, liquid biopsy also plays an important role in treatment and tumor prognosis monitoring, especially in tumor recurrence, metastasis, and drug resistance monitoring, demonstrating its unique advantages. By real-time monitoring of tumor-related biomarker changes, doctors can promptly adjust treatment plans based on liquid biopsy results, thus improving the personalization and precision of treatment. Some common mRNAs or lncRNAs can serve as target molecules for liquid biopsy (42–44). For example, in colorectal cancer, the upregulation of miR-196b-5p in blood has been shown to be closely related to patient resistance to 5-FU chemotherapy (45). The drug resistance mechanism identified via liquid biopsy enables early detection of chemotherapy-resistant patients, allowing clinicians to modify treatment regimens and prevent ineffective therapeutic interventions. In addition, liquid biopsy plays a key role in monitoring tumor recurrence and metastasis. Studies have shown that high expression of CTCs in blood is usually associated with high recurrence rates and poor prognosis. One study on CTC counts on day 1 and day 15 of treatment showed that patients with higher baseline CTC levels had significantly lower overall survival compared to other patients (46). Changes in CTCs not only reflect tumor recurrence but also indicate whether metastasis has occurred. The ctDNA is also an important biomarker in liquid biopsy. Changes in ctDNA can reflect the genetic material released into the blood by tumor cells, providing a reliable method for tumor monitoring. Numerous studies have found that mutations in ctDNA are significantly correlated with patient prognosis. By detecting changes in ctDNA levels, doctors can evaluate patient responses to treatment in real time. If ctDNA levels decrease, it usually indicates that the tumor is being effectively suppressed; if ctDNA levels increase, it may suggest poor treatment response or even the emergence of resistance. For example, in pancreatic cancer patients, mutations in KRAS in ctDNA have been found to correlate with prognosis. The presence of mutations not only suggests a higher risk of early recurrence but is also closely associated with poor overall survival and progression-free survival (47, 48). Another study monitoring ctDNA in pancreatic cancer patients receiving FOLFIRINOX chemotherapy found that in patients with effective treatment, the cfDNA mutation allele fraction (MAF) decreased, while it increased in patients with chemotherapy resistance (49). These findings suggest that liquid biopsy can provide real-time feedback during chemotherapy, helping doctors evaluate whether patients are responding to treatment and detect potential resistance early. Liquid biopsy not only helps evaluate patient responses to single drugs but can also have greater advantages when multiple biomarkers are combined. For example, in the monitoring of pancreatic cancer metastasis, combining several biomarkers in EVs (such as EV-CK18 mRNA, EV-CD63 mRNA, EV-miR-409, cfDNA concentration, and CA19-9) for joint diagnosis shows good clinical results. Studies have shown that these combined markers have an accuracy of 84%, sensitivity of 78%, specificity of 88%, and an AUC value of 0.85 (50). This efficient joint detection strategy greatly improves the sensitivity and accuracy of tumor metastasis monitoring, providing more reliable clinical judgment. In addition, the development of artificial intelligence holds great potential in oncology. It is not only commonly used for tumor image recognition and disease prediction (51–54), but can also be applied to processing the vast and complex output data from liquid biopsies (55, 56).

Based on the bibliometric analysis, we can see the immense potential of liquid biopsy in tumor diagnosis and treatment, especially with the advancement of molecular biology technologies, as research on liquid biopsy gradually transitions from basic science to clinical applications. However, liquid biopsy is still in the early stages of technological development, with existing technologies not yet mature, detection processes being cumbersome, and equipment efficiency limited. These issues hinder its widespread use. For example, the concentrations of CTCs and ctDNA are typically low, affecting detection sensitivity, and contamination may occur during sample extraction and processing, leading to false positives or negatives. Additionally, different sample processing methods can influence the sensitivity and specificity of detection, so improving detection accuracy and efficiency is crucial for further development of liquid biopsy. In addition, the future integration of multi-omics data can provide a more comprehensive molecular profile of tumors, thereby improving diagnostic accuracy and treatment monitoring (57). Artificial intelligence and machine learning algorithms can enhance the analysis of complex liquid biopsy data, enabling better detection of rare biomarkers (58). As an emerging technology, liquid biopsy has yet to establish standardized operational protocols and data analysis processes. To enhance its clinical application value, standardized analysis processes must be developed alongside technological advances (1). In conclusion, the development of liquid biopsy should focus on innovations in new technologies and platforms, strengthen the construction of standardized operating procedures, and promote large-scale clinical trials to ensure its effectiveness and reliability in clinical practice.

This study has several limitations. The literature search and analysis were solely based on WoSCC, which may introduce selection bias. Secondly, due to the lag in citation indexing, the findings may not fully reflect the latest advancements in the field. Additionally, in the analysis of collaborative author contributions, the available data made it difficult to distinguish the specific level of contribution from different authors, potentially affecting the accuracy of collaboration network and research output evaluations. Future research could improve the interpretation of scientific collaboration patterns by integrating multiple databases for cross-validation and incorporating a more granular author contribution annotation system.

## Conclusions

In summary, this article systematically reviews the development history of liquid biopsy technology through bibliometric analysis. The contributions of leading countries in this field reflect a strong research foundation, and international collaboration has further promoted the global dissemination of knowledge. With the increasing number of publications, especially in influential journals, the importance of liquid biopsy in precision medicine is becoming more prominent. In clinical applications, liquid biopsy provides new methods for early cancer screening, treatment evaluation, and resistance monitoring, demonstrating enormous potential and prospects.

## Data Availability

The raw data supporting the conclusions of this article will be made available by the authors, without undue reservation.
